# Effect of NF-κβ inhibitor – dehydroxymethylepoxyquinomicin on iron isomaltoside toxicity toward peritoneal mesothelial cells

**DOI:** 10.1080/0886022X.2024.2304647

**Published:** 2024-01-19

**Authors:** Andrzej Bręborowicz, Kazuo Umezawa

**Affiliations:** a Department of Pathophysiology, Poznan University Medical School, Poznan, Poland; b Collegium Medicum, University of Zielona Góra, Góra, Poland; c Department of Molecular Target Medicine Screening, Aichi Medical University School of Medicine, Nagakute, Japan

Iron supplementation is the standard therapy for patients with uremia requiring renal replacement therapy. In peritoneal dialysis patients, intravenously infused iron diffuses into the peritoneal cavity and induces intraperitoneal inflammation. Peritoneal dialysate-induced mesothelial inflammatory properties are regulated by NFκB, and application of its inhibitor, dehydroxymethylepoxyquinomicin (DHMEQ), modifies these reactions [1].

We evaluated the potential role of DHMEQ on the iron isomaltoside 1000 – Monover^®^ (IIS) induced changes in the functional properties of human peritoneal mesothelial cells (MC) *in vitro*. This study was approved by the Bioethical Committee of the University of Medical Sciences, Poznan, Poland.

MC were cultured in medium M199 with hydrocortisone (1 μg/mL), antibiotics (streptomycin 100 μg/mL and penicillin 100 U/mL), and 10% fetal calf serum (FCS). Experiments were performed on cell monolayers. We studied the effect of IIS on the function of MC in the presence of the NFκB inhibitor – DHMEQ at a concentration of 10 µg/mL, which is nontoxic to MC [1]. Concentration of IIS used in the study was similar to the dialysate level of iron found in the peritoneal dialysate after i.v. infusion of IIS [2].

The following experimental groups were studied:

Control group – culture medium;
IIS group – culture medium plus IIS 15 µg/dL;
DHMEQ group – culture medium plus DHMEQ 10 µg/mL;
IIS – DHMEQ group – culture medium plus IIS 15 µg/dL and DHMEQ 10 µg/mL.

In groups 3 and 4, before the start of the experiment, MC were preincubated for 6 h in medium containing DHMEQ (10 µg/mL). In all groups, the cells were incubated for 24 h in medium. The experiments were repeated nine times using MC obtained from different donors.

At the end of the incubation period, supernatants were collected to measure MC secretory activity. The levels of the following substances were measured using ELISA (DuoSet Immunoassay; R&D Systems, Minneapolis, MN): interleukin 6 (IL6), tissue plasminogen activator (tPA), plasminogen activator inhibitor-1 (PAI-1), and transforming growth factor-β1 (TGFβ1). To quantify the cell number, the cells were harvested from the wells with trypsin 0.05% – EDTA 0.02% solution and counted using a hemocytometer. Protein concentrations in cell lysates were measured using the Lowry method [3]. Intracellular iron in MC was measured using a colorimetric ferrozine-based method [4]. The viability of the cell monolayers was measured using an MTT assay (Abcam, Cambridge, UK). Free radicals generated within the cells were measured after 45 min of incubation at 37 °C with a 2′7′-dichlorodihydrofluorescein diacetate probe. Fluorescence of the cell lysates was measured using a fluorimeter at a wavelength of 485 nm for excitation and 535 nm for emission.

Total RNA was isolated from the MC monolayers, after exposure to the studied media (groups 1–4), using the ReliaPrep™ RNA Cell Miniprep System (Promega, Madison, WI) and, treated with DNase I using DNA-free DNase Treatment and Removal Reagent (Thermo Fisher Scientific, Waltham, MA). RNA was reverse-transcribed into cDNA using a Transcriptor First Strand cDNA Synthesis Kit (Roche, Basel, Switzerland). Relative mRNA levels of the four genes:

IL6 [F: ATGAACTCCTTCTCCACAAGC; R: GTTTTCTGCCAGTGCCTCTTTG];
t-PA [F: CAGCCAGGAAATCCATGCCC; R: GCCATGACTGATGTTGCTGG];
PAI-1 [F: TGCTGGTGAATGCCCTCTACT; R: CGGTCATTCCCAGGTTCTCTA];
TGF-β1 [F: TGGAAATCAATGGGATCAGTC; R: GAGCAAGTGCTTGGTATGG].

were studied in triplicates for each experiment and normalized to the levels of an internal house-keeping gene: glyceraldehyde-3-phosphate dehydrogenase (GAPDH) [F: TTCGTCATGGGTGTGAACC; R: GATGATGTTCTGGAGAGCCC]. Relative gene expression was calculated using the 2^–ΔΔCt^ method [5].

Total protein and collagen synthesis was measured in MC exposed to the studied media supplemented with β-aminopropionitrile (50 μg/mL), l-ascorbic acid (50 μg/mL) and ^3^H-proline (5 μCi/mL). After 24 h of incubation, cells were lysed by repeated freezing and thawing. The supernatants, and cell lysates collected from each well were divided into two equal portions (2 × 0.5 mL), which were mixed (1:1, v/v) as follows:

- Hanks’ solution with N-ethylmaleimide (2.5 mM/mL);
- Hanks’ solution containing N-ethylmaleimide (2.5 mM/mL) and collagenase 0.2 mg/mL).

The mixtures were incubated for 4 h at 37 °C, and the proteins in each sample were precipitated with 10% trichloroacetic acid (TCA). After centrifugation and removal of the supernatant, the precipitate was washed twice with TCA and lysed at 4 °C in 0.1 N NaOH. The radioactivity of cell lysates was measured using a β-liquid scintillation counter. The radioactivity of sample A reflected total protein synthesis, and the difference in radioactivity between samples A and B was used as an index of collagen synthesis.

Results are presented as mean ± SD. Data analysis was performed using the nonparametric Kruskal–Wallis test with the *post hoc* analysis between the groups.

Treatment of MC cells with the studied media did not affect their viability, as confirmed by the results of the MTT test: (% of control) 96 ± 5 in IIS group, ns; 97 ± 4 in DHMEQ group, ns; 97 ± 5 in IIS + DHMEQ group, ns. In the cells exposed for 24 h to media with IIS, or with IIS + DHMEQ, intracellular iron content was increased as compared to cells not treated with iron by 77% and 81%, respectively (*p* < .001). IIS treatment resulted in increased intracellular oxidative stress (+118%, *p* < .001); however, when the cells were simultaneously treated with DHMEQ, the increase in intracellular free radicals was smaller (+40%, *p* < .01).

**Figure 1.**
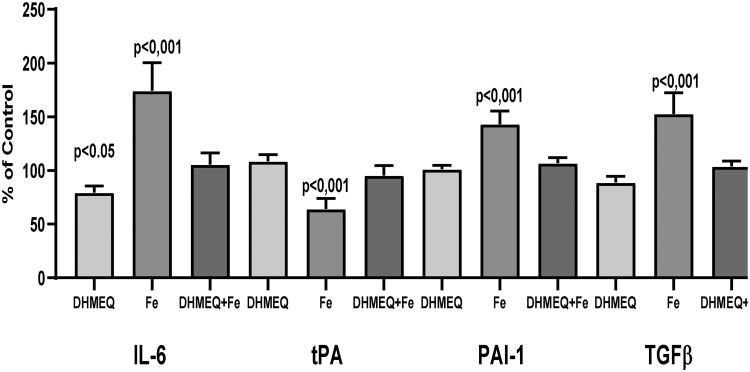
Expression of genes regulating expression of IL6, tPA, PAI-1, and TGFβ in mesothelial cells cultured in medium supplemented with DHMEQ 10 μg/mL (DHMEQ), IIS 15 μg/dL (Fe) or DHMEQ 10 μg/mL plus IIS 15 μg/dL (DHMEQ + Fe). Results are presented as % of control when cells were cultured in standard medium.

**Figure 2.**
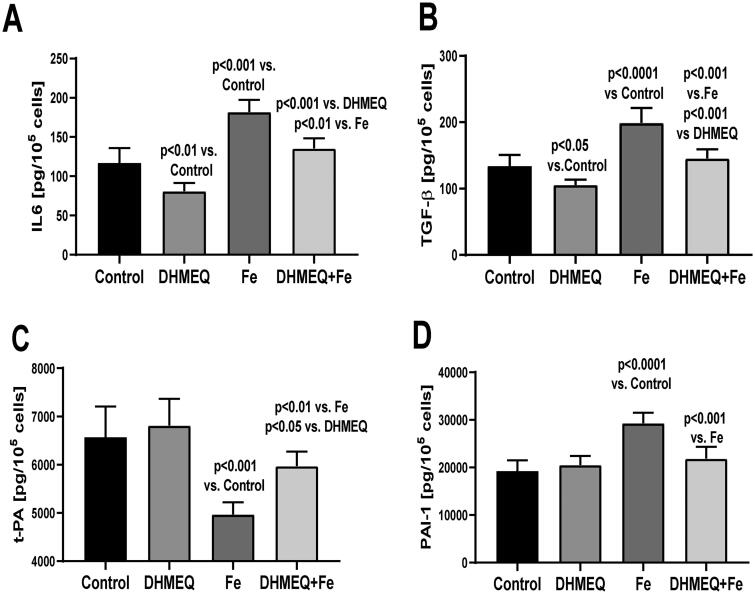
Secretion of IL6 (A), TGFβ (B), t-PA (C), and PAI-1(D) in mesothelial cells cultured in standard medium (control), medium supplemented with DHMEQ 10 μg/mL (DHMEQ), IIS 15 μg/dL (Fe), or DHMEQ 10 μg/mL plus IIS 15 μg/dL (DHMEQ + Fe) (*p* < .001) (*n* = 9).

Exposure of cells to a medium supplemented with IIS caused changes in gene expression: an increase in IL6 (+74%, *p* < .001), PAI-1 (+43%, *p* < .01), and TGFβ1 (+53%, *p* < .001), whereas the expression of the tPA gene was reduced (–36%, *p* < .01). Simultaneous exposure to DHMEQ significantly reduced iron-induced changes in gene expression (Figure 1). Changes in gene expression were reflected by the modified cellular secretory activity (Figure 2). IIS stimulated the secretion of IL6 (+56%, *p* < .001), but in the presence of DHMEQ, IL6 synthesis was lower (–32%, *p* < .01) (Figure 2(A)). IIS stimulated the release of TGFβ1 from MC (+49%, *p* < .001), and in the presence of DHMEQ, secretion of TGFβ1 was lower (–27%, *p* < .001) than in cells treated with IIS alone. When DHMEQ was used as the only supplement to the medium, the synthesis of TGFβ1 was lower than that in the control group (–21%, *p* < .05) (Figure 2(B)). The synthesis of t-PA was reduced in cells exposed to IIS (–25%, *p* < .001) and this effect was weaker (–12%, *p* < .01) when DHMEQ was used simultaneously (Figure 2(C)). In the presence of IIS, synthesis of PAI-1 increased by 51% (*p* < .001), and no such effect was observed when DHMEQ was used (Figure 2(D)).

The synthesis of total proteins was comparable in all the studied groups. In the presence of IIS, collagen synthesis was higher (+45%, *p* < .001) than in the control group. However, when IIS was used together with DHMEQ, collagen synthesis was reduced (–25%, *p* < .001) compared to the effect of IIS alone.

The presented results show that iron induces intracellular stress in MC via NFκβ system, not only through their proinflammatory phenotype [6] but also through profibrotic activity due to increased TGFβ synthesis. Proinflammatory changes reflected by increased secretion of IL6 may enhance the fibrotic changes in the peritoneum due to increased deposition of fibrin within peritoneum caused by reduced tPA and increased PAI-1 synthesis. The use of the NFκB inhibitor DHMEQ reduces these changes. Further studies are required to prove the effectiveness of DHMEQ in an *in vivo* peritoneal dialysis scenario.
